# Detection of the Anaerobic Threshold in Endurance Sports: Validation of a New Method Using Correlation Properties of Heart Rate Variability

**DOI:** 10.3390/jfmk6020038

**Published:** 2021-04-26

**Authors:** Bruce Rogers, David Giles, Nick Draper, Laurent Mourot, Thomas Gronwald

**Affiliations:** 1 College of Medicine, University of Central Florida, 6850 Lake Nona Boulevard, Orlando, FL 32827-7408, USA; 2 Lattice Training Ltd., Chesterfield S41 9AT, UK; dave@latticetraining.com; 3 School of Health Sciences, College of Education, Health and Human Development, University of Canterbury, 8041 Christchurch, New Zealand; nick.draper@canterbury.ac.nz; 4 EA3920 Prognostic Factors and Regulatory Factors of Cardiac and Vascular Pathologies, Exercise Performance Health Innovation (EPHI) Platform, University of Bourgogne Franche-Comté, 25000 Besançon, France; laurent.mourot@univ-fcomte.fr; 5 Division for Physical Education, National Research Tomsk Polytechnic University, Lenin Ave, 30, 634050 Tomsk Oblast, Russia; 6 Department of Performance, Neuroscience, Therapy and Health, Faculty of Health Sciences, MSH Medical School Hamburg, University of Applied Sciences and Medical University, Am Kaiserkai 1, 20457 Hamburg, Germany; thomas.gronwald@medicalschool-hamburg.de

**Keywords:** autonomic nervous system, HRV, intensity distribution, endurance training

## Abstract

Past attempts to define an anaerobic threshold (AnT) have relied upon gas exchange kinetics, lactate testing and field-based evaluations. DFA a1, an index of heart rate (HR) variability (HRV) fractal correlation properties, has been shown to decrease with exercise intensity. The intent of this study is to investigate whether the AnT derived from gas exchange is associated with the transition from a correlated to uncorrelated random HRV pattern signified by a DFA a1 value of 0.5. HRV and gas exchange data were obtained from 15 participants during an incremental treadmill run. Comparison of the HR reached at the second ventilatory threshold (VT2) was made to the HR reached at a DFA a1 value of 0.5 (HRVT2). Based on Bland–Altman analysis and linear regression, there was strong agreement between VT2 and HRVT2 measured by HR (*r* = 0.78, *p* < 0.001). Mean VT2 was reached at a HR of 174 (±12) bpm compared to mean HRVT2 at a HR of 171 (±16) bpm. In summary, the HR associated with a DFA a1 value of 0.5 on an incremental treadmill ramp was closely related to that of the HR at the VT2 derived from gas exchange analysis. A distinct numerical value of DFA a1 representing an uncorrelated, random interbeat pattern appears to be associated with the VT2 and shows potential as a noninvasive marker for training intensity distribution and performance status.

## 1. Introduction

The identification of physiologic indicators representing breakpoints involved in endurance exercise intensity is of vital importance for both performance monitoring and exercise intensity distribution [1,2]. In the classic three-zone model, the intensity boundaries are defined by either certain gas exchange parameters or blood lactate determination [3,4]. The lowest intensity zone is felt to be delimited by the first ventilatory (VT1) or lactate threshold (LT1) and is described as representing an aerobic threshold (AeT). The highest intensity zone, encompassing work rates above the second ventilatory (VT2) or lactate thresholds (LT2), is described as an anaerobic threshold (AnT) and is felt to be unsustainable for long durations [5]. Although the VT2 appears to be characterized as the first systematic increase in the ventilatory equivalent of CO_2_ or the first decrease in the expiratory fraction of CO_2_ during heavy exercise, the LT2 has had several alternate definitions [4,6]. These include the exercise intensity associated with reaching a fixed lactate of 4 mmol/l, various calculations and exponential computations models and a maximal lactate steady state (MLSS) [4,6]. To complicate matters further, many of these concepts can yield different results depending on incremental ramp progression, stage protocol or even expert visual interpretation [2,6,7]. In addition, both gas exchange and lactate testing require specialized equipment, operators, can be invasive and are costly. An initially promising approach to AnT measurement, the identification of deoxyhemoglobin breakpoints of locomotor muscle [8,9], has unfortunately been hindered by the abandonment of commercial products due to poor financial outcomes. In an effort to identify the AnT noninvasively, field-based tests have been devised with the functional threshold power (FTP) evaluation being a popular example [10]. However, results have been shown to vary depending on warmup protocol and test procedures [11,12,13]. In addition, the FTP is dependent on motivation, individual pacing strategy [14] and by its definition, physically exhausting. Another maximal effort test, critical power (CP) has also received attention as a means of AnT delineation [15]. However, discrepancy between the CP, FTP, VT2 and MLSS [5,14,16] has been debated and concordance is unclear. Therefore, agreement of performance based tests of the AnT can be variable, and each has a potential detrimental impact on an athlete’s training intensity distribution strategy. Given these considerations, a search for alternative, objective, noninvasive methods for determining the AnT seems reasonable.

Previous analysis of the dynamic change in heart rate (HR) variability (HRV) during exercise has also shown potential for demarcation of threshold boundaries, particularly the AeT [17]. The mechanism affecting HRV during exercise is felt to be related to alteration in autonomic balance to the cardiac sinoatrial pacemaker center. As intensity rises, there is a withdrawal of parasympathetic influence and augmentation of sympathetic stimulation [18]. Conventional HRV indexes such as standard deviation of normal-to-normal RR intervals (SDNN), high-frequency (HF) power and standard deviation 1 from Poincaré plot analysis (SD1) can be used to identify the AeT during incremental exercise ramps by observing at what exercise intensity a nadir HRV value occurs [19,20,21]. Since these particular indexes reach their lowest value at the AeT, they are not felt to be helpful for AnT delineation given the loss of any further dynamic range. In contrast, a nonlinear index of fractal correlation properties called alpha1 of Detrended Fluctuation Analysis (DFA a1) possesses a much wider dynamic range [22,23]. This index is based on both the fractal nature and “correlation pattern” of a series of cardiac beat to beat intervals over time [17]. Fractal behavior in relation to HRV can be described as degrees of self-similarity between RR interval fluctuations over different time scales and allows a distinction of the fractal character of the physiological signal between fractional Brownian motion (fBm: DFA a1 > 1.0) and fractional Gaussian noise (fGn: DFA a1 < 1.0) [24,25]. Analogously, this conception allows the determination of different alterations of complexity in HR time series, either toward rigid order with increasing correlation properties or toward disorder with decreasing correlation properties [26]. At low exercise intensity, DFA a1 values are usually in a well-correlated fractal range near or above 1.0. As intensity rises, DFA a1 passes 0.75 at the AeT [27] and continues to drop through the 0.5 range associated with uncorrelated random behavior of interbeat pattern, finally to drop below 0.5 representing an anticorrelated range at the very highest work rates, which could be seen as a protective feedback and stabilizing mechanism where interactions and/or coordination of subsystems fail before the whole system fails [23,26,28]. Advantages of DFA a1 for intensity monitoring also revolve around its dimensionless nature, which makes calibration to other internal load parameters such as gas exchange or lactate unnecessary. In other words, a HR or VO_2_ associated with a DFA a1 value of 0.75 corresponds to an intensity near the AeT/VT1 in a wide spectrum of individuals and was referred to as the HRVT (heart rate variability threshold) [27].

Since DFA a1 still possesses reasonable dynamic range at intensities above the AeT, the question arises whether another numerical threshold exists corresponding to the AnT. From a mathematical standpoint, a DFA a1 value of 0.5 is of interest as a breakpoint for a high-intensity physiologic process. A value of 0.5 denotes a loss of correlation and fractal patterns in the beat to beat cardiac time series. To better understand the concept of “correlation properties”, a comparison to a random walk can be used [25,28,29]. During a hypothetical random walk, at each advancing step, the walker can choose to go either left or right. If the choice the walker makes is not random but based on the pattern (series of left or right choices) of what went beforehand, the pattern is described as being correlated (DFA a1 about 1.0), since the future pattern is based on the past sequence. However, if each new step is taken with equal, random chances of left or right, an “uncorrelated” pattern exists (DFA a1 about 0.5). Additionally, from an observational perspective, DFA a1 values well below 0.5 occur at the termination of maximal exercise ramps, leading to the suspicion that the 0.5 value may have significance as a high-intensity boundary near the AnT [23]. Therefore, the intent of this study is to investigate whether reaching a DFA a1 of 0.5 during an incremental exercise ramp is associated with the VT2, a ventilatory marker of the AnT. Since both artifact correction and recording device bias could be an issue with DFA a1 precision [30], high quality ECG tracings will be utilized for HRV interpretation. In addition, given practical considerations for training prescription in sports and rehabilitation, only HR measurements will be compared given the excellent agreement between the HR and VO_2_ relationship during exercise [31].

## 2. Methods

### 2.1. Participants

Seventeen male recreational runners aged 19 to 52, without previous medical history, current medications or physical difficulties were tested. Participants were informed of the possible testing risks and institutionally approved consent was given. Approval for the research was granted by the University of Derby, UK (LSREC_1415_02) and followed the principles of the Declaration of Helsinki. Runners did not consume caffeine, alcohol or any stimulant for the 24 h before testing. There was no tobacco usage. Background data for each participant including, age, body weight, and training volume in hours per week are presented in Table 1 and were also published in an earlier work [27]. Testing was performed in the afternoon and at least 3 h after food (with no set diet). No intense activity was performed the day prior to the test. Two participants with excessive cardiac ectopy (frequent atrial premature beats and atrial trigeminy) during testing were excluded from HRV analysis.

### 2.2. Exercise Protocol

A motorized treadmill (Woodway, Birmingham, UK) was used for an incremental maximal cardiopulmonary exercise test for all runners. The treadmill was set for the Bruce protocol with increases in speed and inclination from 2.7 km/h at ten percent grade, increasing by 1.3 km/h and two percent grade every 3 min until exhaustion. A fan was used for cooling. Room temperature was approximately 24 °C for all tests.

Gas Exchange Testing and Calculation of the AnTGas exchange kinetics were recorded continuously using a breath to breath metabolic cart (Metalyzer; Cortex Biophysik GmbH, Leipzig, Germany), with a Polar H7 (Polar Electro Oy, Kempele, Finland) wirelessly paired to the Metalyzer cart for the purpose of HR recording concurrent with gas exchange data. Heart rate corresponded to each reported breath to breath data point. Assessment of the VO_2_ over time relationship was performed to determine any significant plateau of the VO_2_ curve for estimation of VO_2_ linearity. VT2 associated HR was determined by Oxynet [32,33], a convolutional neural network previously shown to have excellent agreement (average mean absolute error = 6.1%, *r*  =  0.99) with manually derived results especially in individuals with medium to high aerobic fitness levels. Raw gas exchange data were uploaded to the Oxynet web app (http://oxynetresearch.promfacility.eu (accessed on 20 February 2021)) followed by a download of results.

### 2.3. RR Measurements and Calculation of DFA a1 Derived Threshold

Each participant’s ECG/RR times series was recorded with a 3-lead ECG (MP36; Biopac Systems Ltd., Essen, Germany) with a sampling rate of 1000 Hz. Electrodes were placed in the CM5 distribution after appropriate skin cleaning. MP36 test data were saved as acq files. ECG data for each participant were imported into Kubios HRV Software (Version 3.4.3, Biosignal Analysis and Medical Imaging Group, Department of Physics, University of Kuopio, Kuopio, Finland). Kubios preprocessing settings were set to the default values including the RR detrending method which was kept at “Smoothn priors” (Lambda = 500). For DFA a1 estimation, the root mean square fluctuation of the integrated and detrended data is measured in observation windows of different sizes. The data are then plotted against the size of the window on a log–log scale [34]. The scaling exponent represents the slope of the line, which relates (log) fluctuation to (log) window size. DFA a1 window width was set to 4 ≤ *n* ≤ 16 beats. Visual inspection of the entire test recording was done to determine sample quality, noise, arrhythmia, and missing beat artifact. As mentioned above, two participants with excessive atrial ectopy were excluded from analysis. The RR series of the included participants was then corrected by the Kubios “automatic method” [35] and relevant HRV results exported as text files for further analysis. Percent artifact occurring during threshold interpretation segments was below 5%.

**Figure 1:**
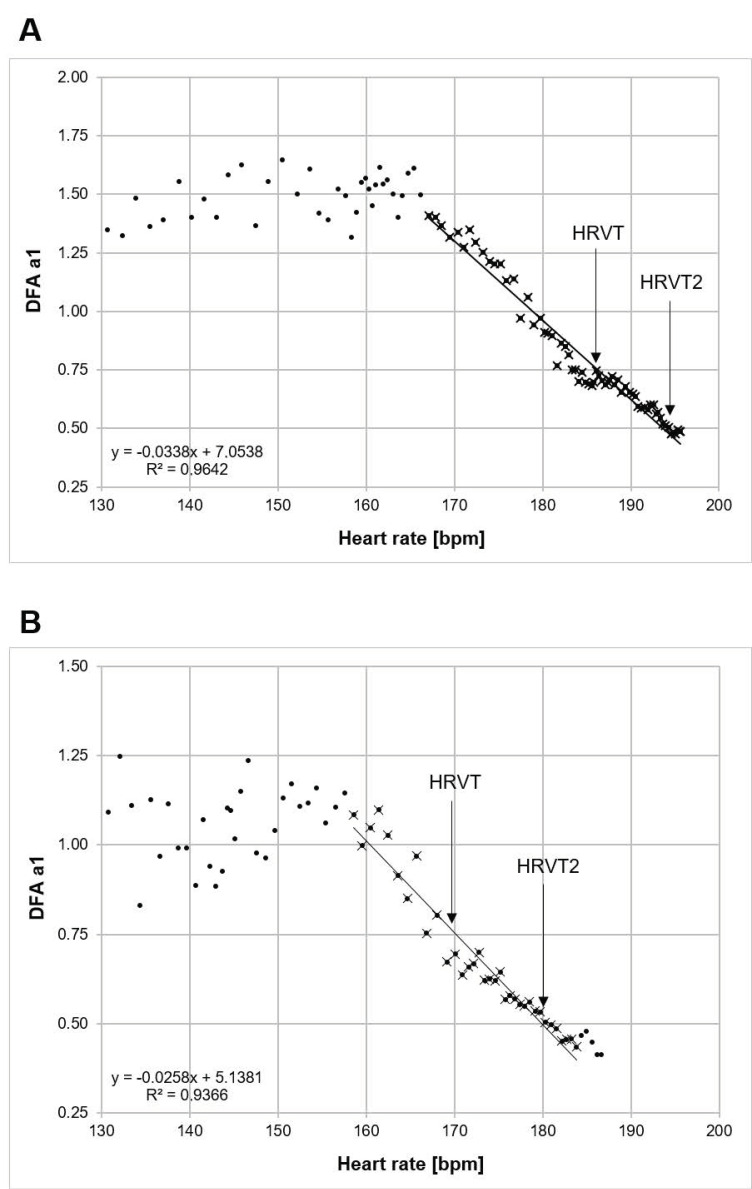
Individual examples of calculation methods for both HRVT and HRVT2. A line is drawn through the straight section of the plot of DFA a1 vs. HR. HRVT is at the intersection of DFA a1 = 0.75 and HRVT2 is at the intersection of DFA a1 = 0.5. (**A**) Participant with a VO_2MAX_ of 72 mL/kg/min, VT2 at 192 bpm and HRVT2 at 194 bpm. (**B**) Participant with a VO_2MAX_ of 58 mL/kg/min, VT2 at 179 bpm and HRVT2 at 180 bpm. Points with X are used for linear regression.

The following procedure was used to indicate at what level of running intensity HR the DFA a1 would cross a value of 0.5: DFA a1 was calculated from the incremental exercise test RR series using 2 min time windows with a recalculation every 5 s throughout the test. Two-minute time windowing was chosen based on the minimal required RR interval calculations by Chen et al. [36]. Plotting of DFA a1 vs. HR was then performed. Inspection of the DFA a1 relationship with HR generally showed a reverse sigmoidal curve, with a stable area above 1.0 at low work rates, a rapid, near linear drop reaching below 0.5 at higher intensity, then flattening without major change. Linear regression was done on the subset of data consisting of the rapid, near linear decline from values close to 1.0 (correlated) to approximately 0.5 (uncorrelated) or below if the values continued in a straight fashion. The HR where DFA a1 reached 0.5 was calculated based on the regression equation from that linear section (Figure 1).

## 3. Statistics

Statistical analyses were performed for the variables HR at VT2 derived from gas exchange testing and HR at DFA a1 of 0.5 (HRVT2). Standard statistical methods were used for the calculation of means and standard deviations (SD). Normal distribution of data was checked by Shapiro–Wilk’s test. The agreement against the VT2 HR was assessed using linear regression, Pearson’s r correlation coefficient, coefficient of determination (R^2^), standard error of estimate (SEE) and Bland–Altman plots with limits of agreement [37]. The size of Pearson’s r correlations were evaluated as follows: 0.3 ≤ r < 0.5 low; 0.6 ≤ r < 0.8 moderate and r ≥ 0.8 high [38]. The paired t-test was used for comparison of VT2 HR vs. HRVT2 HR. For all tests, the statistical significance was accepted as *p* ≤ 0.05. Analysis was performed using Microsoft Excel 365 with Real Statistics Resource Pack software (Release 6.8) and Analyse-it software (Version 5.66).

## 4. Results

**Figure 2:**
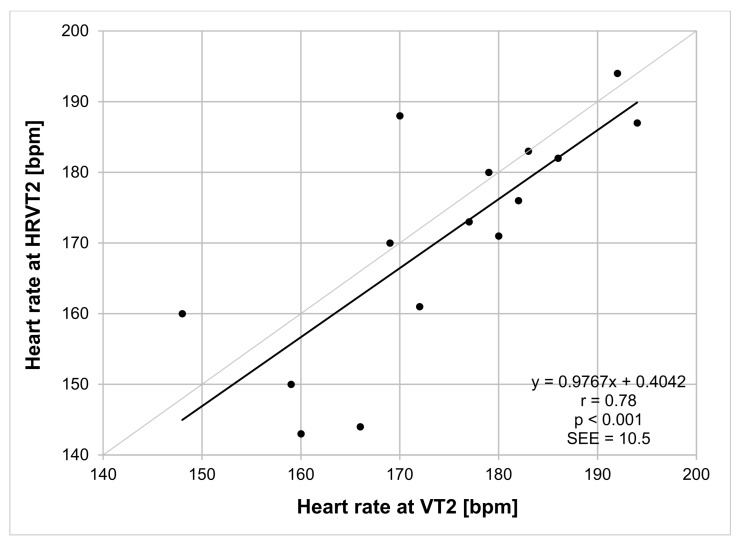
Regression plot analysis VT2 HR vs. HRVT2 HR for all participants. Bisection line in light gray. SEE: standard error of estimate; r: Pearson’s correlation coefficient.

**Figure 3:**
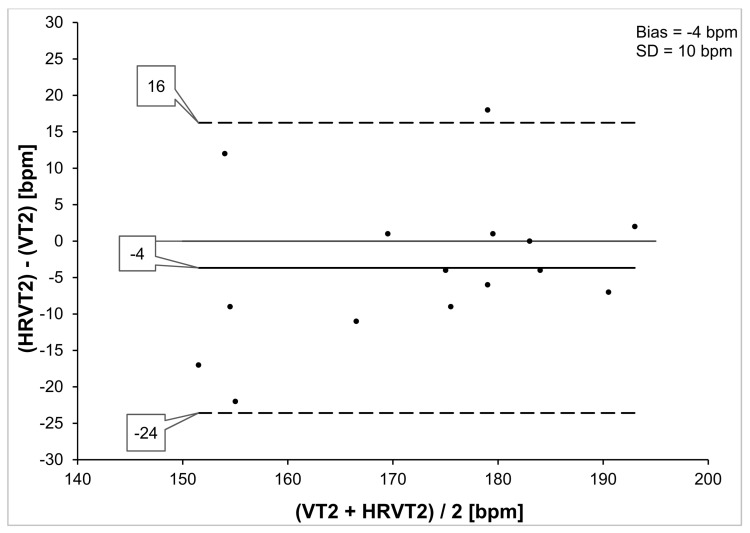
Bland–Altman analysis VT2 HR vs. HRVT2 HR for all participants. Center line represents the mean difference between each paired value, the top and bottom lines are 1.96 standard deviations from the mean difference.

VO_2MAX_ varied considerably among participants, ranging between 41 and 74 mL/kg/min. VT1 was reached at HRs between 108 and 183 bpm [27]. Oxynet-derived HR at VT2 showed a mean value of 174 (±12) bpm compared with a mean HRVT2 HR of 171 (±16) bpm (*p* = 0.18) for all participants (Table 1). Regression analysis for VT2 HR vs. HRVT2 HR showed significant correlation (*p* < 0.001) with Pearson’s r = 0.78, R^2^ = 0.60 and SEE = 10.5 bpm (Figure 2). Bland–Altman evaluation of VT2 HR vs. HRVT2 HR is shown in Figure 3 with a mean bias of −4 (±10) bpm and LOA from −24 to +16 bpm.

## 5. Discussion

The purpose of this study was to examine whether DFA a1, a HRV index of fractal correlation properties would exhibit an uncorrelated pattern at the AnT derived by gas exchange data, a boundary separating sustainable from unsustainable exercise intensity [5]. In a previous investigation, it was shown that a DFA a1 value of 0.75, which represents a midpoint between well-correlated and uncorrelated patterns, was associated with the AeT as measured by VT1 [27]. In the present report, the HR reached at a DFA a1 of 0.5 was closely related to the HR reached at the AnT as measured by the VT2 during a treadmill running ramp. Multiple prior studies have shown similar behavior during incremental exercise with DFA a1, declining past the 0.75 mark with mild to moderate intensity then surpassing 0.5 during the highest work rates attained [23]. Prior to this report no attempt has been made to determine if the AnT is associated with a particular DFA a1 value using a method validation comparison. Strengths of this study include RR recording done by a research-grade ECG device containing few artifacts and the inclusion of recreational runners with a wide age range and performance spectrum. In addition, VT2 was computed by a validated neural network system utilizing the raw gas exchange data, eliminating any observer error or bias [32,33].

The results obtained here show good agreement between the HR derived from the HRVT2 and the HR associated with VT2 obtained through Oxynet analysis. This was supported by comparison of mean HR parameters by paired t testing, Pearson’s correlation coefficient and Bland–Altman analysis. Although participants had variable differences in HR concordance, the results are clinically meaningful in the context of the reported agreement of other surrogate markers. Both the mean difference of −4 bpm and the LOA seen here are of similar magnitudes to that of a comparison of the MLSS and FTP [12] as well as the muscle oxygen desaturation breakpoint association to the MLSS [8].

The question as to why a DFA a1 of 0.5 could be the area of interest for an anaerobic breakpoint should be discussed. Prior studies have shown DFA a1 to drop past the uncorrelated value of 0.5 to an anticorrelated range at near maximal attained work rates [23]. Recently, a study by Naranjo-Orellana et al. [39] showed that during constant-intensity exercise performed over a 5 min span at the VT2, a DFA a1 value of 0.48 (±0.11) was seen, very similar to our results. Therefore, from an observational perspective, it is not unreasonable to look for the AnT to occur near the 0.5 value. Perhaps most importantly, a DFA a1 of 0.5 specifically represents a transition from an uncorrelated random to an anticorrelated pattern in HR time series [28]. Viewpoints regarding the significance of correlation properties can revolve around practical aspects (empirically validated breakpoints such as 0.75) but can also be considered from a network physiology standpoint [26]. The later concept entails the notion that fractal correlation properties of HRV depend upon “organismic demand”, a model of multiple neuromuscular, biochemical, peripheral and central nervous system inputs [17]. In this framework, hypothetical acute physiologic responses and cardiocirculatory advantages may lie behind the changes seen in correlation patterns due to increasing exercise intensity and/or overall organismic demands. Depending on the internal load situation, the correlation properties of HR time series change to best suit the current and perhaps even the anticipated requirements as an optimization and/or stabilization strategy [28,40,41]. Therefore, the anticorrelated behavior during very high-intensity exercise could be interpreted in the sense of progressive segregation and centralization or “mechanization” of a complex (open) biological system as proposed by von Bertalanffy [42,43] and could indicate a maximum energy flux at the cost of cardiovascular self-regulation, which may reduce the adaptability to further perturbations and ultimately endangers the integrity of the overall system [44,45,46]. Thus, every fluctuation is corrected immediately in the opposite direction by a dominant attractor [29], e.g., performance attracter, as stated by Gronwald et al. [17], which results in an anticorrelated signal pattern [28]. This organismic regulatory withdrawal may also be interpreted as a loss of systemic integrity in the sense of a hazardous situation for homeostasis [47], which may only be tolerated for a short period of time.

## 6. Limitations and Future Directions

A potential problem with using HRV-related indexes to determine a physiologic threshold boundary involves the quality and precision of the RR time series [30,48]. Since the rate of missed beat artifact rises with increasing exercise intensity [49], excess amounts of artifact correction can affect DFA a1 resulting in bias and erroneous estimation of threshold values. Common methods of artifact correction may produce a positive proportional bias in DFA a1, predominantly affecting values 0.5 and below, especially at artifact rates above 5% [30]. No participant in the cohort examined here exceeded that limit. In addition, it is also possible that recording device bias may occur leading to results that differ from those of high resolution ECG monitoring. In view of these issues, further validation of this approach is recommended with commonly used consumer monitoring devices and typical artifact levels. However, if these issues can be resolved, analysis of DFA a1 over the course of an exercise ramp may provide both aerobic and anerobic threshold boundaries for the purpose of endurance sport training intensity distribution. Since all participants in this study were male, evaluation in females is important for widespread future usage. It is also unclear what the effects of athlete status (elite, recreational, inactive), ramp protocol (short vs. long, slope), exercise type (cycling vs. running vs. XC skiing), food intake, caffeine and recent high-intensity exercise would have on the HRVT2. Future use of this approach in the study of exercise training interventions may also be of interest. Though no data currently exist, following DFA a1 over the course of an intervention protocol could be of interest as a surrogate marker for AnT-related performance improvement. An intriguing thought centers on the methods used to obtain each AnT-related metric in this study, one from a convolutional neural network of gas exchange parameters and the other from a relatively simple mathematical relationship of HRV. Although at this time Oxynet relies purely on respiratory parameters, it could be of interest to determine whether adding DFA a1 HRVT-related measurements would improve final accuracy. Lastly, as a DFA a1 reaching 0.5 is sufficient for boundary determination, exercise efforts to exhaustion such as the FTP or CP can be avoided, both for health-related and exercise intensity distribution purposes.

## 7. Conclusions

Nonlinear heart rate variability analysis during an incremental treadmill run demonstrated that the heart rate reached at the second ventilatory threshold was closely associated with that of the heart rate associated with a DFA a1 of 0.5 in a population of recreational athletes. This DFA a1 value represents a distinct mathematical breakpoint in the cardiac interbeat series, from a correlated pattern seen with light to moderate exercise intensity to an uncorrelated, random pattern of heart rate time series occurring at the point of an unsustainable work rate. Although promising, additional study and verification in females, other exercise modalities, recording devices and disease states are recommended. Since this method may not require testing to exhaustion, application to athletes avoiding maximal stress during a given training cycle and to those unable/unsuitable to undergo maximal intensity exercise may be possible. In combination with evidence of a DFA a1 of 0.75 representing the aerobic threshold boundary, a comprehensive solution for training boundary demarcation using only heart rate variability may soon be achieved.

## Figures and Tables

**Table 1:** Age, training volume (TV), bodyweight (BW), maximal oxygen uptake (VO_2MAX_), Oxynet-derived HR at VT2 and HR at HRVT2 for all participants.

Nr.	Age [yrs]	TV [hrs/wk]	BW [kg]	VO_2MAX_ [mL/kg/min]	VT2 [bpm]	HRVT2 [bpm]
1	19	3–6	82	58	179	180
2	19	3–6	82	57	183	183
3	20	3–6	82	47	194	187
4	22	1–3	73	45	170	188
5	23	>6	77	71	148	160
6	24	3–6	69	64	166	144
7	24	>6	65	54	177	173
8	24	3–6	76	47	182	176
9	25	>6	78	54	169	170
10	26	>6	69	72	192	194
11	30	1–3	92	46	160	143
12	30	>6	73	74	172	161
13	32	1–3	65	49	186	182
14	36	>6	75	57	180	171
15	50	3–6	94	41	159	150
Mean (SD)	27 (±8)	-	77 (±8)	56 (±10)	174 (±12)	171 (±16)

## Data Availability

The raw data supporting the conclusions of this article will be made available by the authors, without undue reservation.
